# Supplementary material for: Identification and functional analysis of non-coding regulatory small RNA FenSr3 in Bacillus amyloliquefaciens LPB-18
Source: PeerJ. 2023 May 15;11:e15236. doi: 10.7717/peerj.15236 (PMC10194069; doi:10.7717/peerj.15236)
Supplement: Supplemental Information 4 [file peerj-11-15236-s004.zip › KO/CK-vs-T1_map/map04950.html]

KEGG PATHWAY: Maturity onset diabetes of the young - Reference pathway


|  |  |
| --- | --- |
| **Maturity onset diabetes of the young - Reference pathway** |  |

[
Pathway menu
| Organism menu
| Pathway entry
| Show description
| User data mapping
]

About 2-5% of type II diabetic patients suffer from a monogenic disease with autosomal dominant inheritance. This monogenic form of type II diabetes is called maturity onset diabetes of the young (MODY).
We now know that MODY is caused by heterozygous mutations in at least five genes encoding transcription factors: HNF4alpha (MODY1), HNF1alpha (MODY3), PDX1 (MODY4), HNF1beta (MODY5) and NEUROD1 (MODY6). MODY2, which is so far the only subtype not related to a transcription factor, is caused by mutations in the glucokinase gene. Mutations of MODY transcription factor genes lead to abnormal expression of genes involved in pancreatic islet development and metabolism.

|  |  |  |
| --- | --- | --- |
| Reference pathway | 184% 150% 122% 100% 82% 67% 55% | 图片下载 |
